# Characterization and Functional Studies of a Novel Depolymerase Against K19-Type *Klebsiella pneumoniae*

**DOI:** 10.3389/fmicb.2022.878800

**Published:** 2022-06-22

**Authors:** Yunfen Hua, Yongqin Wu, Minjie Guo, Ruijing Ma, Qingchuan Li, Zheyuan Hu, Hongrui Chen, Xingyu Zhang, Hui Li, Qingtian Li, Ping He

**Affiliations:** ^1^College of Pharmaceutical Sciences, Zhejiang University of Technology, Hangzhou, China; ^2^Department of Immunology and Microbiology, Shanghai Jiao Tong University School of Medicine, Shanghai, China; ^3^Department of Nanoengineering, University of California, San Diego, La Jolla, CA, United States; ^4^NHC Key Laboratory of Parasite and Vector Biology, National Institute of Parasitic Diseases, Chinese Center for Disease Control and Prevention, Shanghai, China; ^5^Department of Laboratory Medicine, Ruijin Hospital, Shanghai Jiao Tong University School of Medicine, Shanghai, China

**Keywords:** carbapenem-resistant *Klebsiella pneumoniae*, bacteriophage, depolymerase, capsular typing, anti-infection of CRKP

## Abstract

Carbapenem-resistant *Klebsiella pneumoniae* (CRKP), a pathogen that causes severe nosocomial infections and yields a high mortality rate, poses a serious threat to global public health due to its high antimicrobial resistance. Bacteriophages encode polysaccharide-degrading enzymes referred to as depolymerases that cleave the capsular polysaccharide (CPS), one of the main virulence factors of *K. pneumoniae*. In this study, we identified and characterized a new capsule depolymerase K19-Dpo41 from *K. pneumoniae* bacteriophage SH-KP156570. Our characterization of K19-Dpo41 demonstrated that this depolymerase showed specific activities against K19-type *K. pneumoniae*. K19-Dpo41-mediated treatments promoted the sensitivity of a multidrug-resistant K19-type *K. pneumoniae* strain to the bactericidal effect of human serum and significantly increased the survival rate of *Galleria mellonella* infected with K19-type *K. pneumoniae*. Our results provided strong primary evidence that K19-Dpo41 was not only effective in capsular typing of K19-type *K. pneumoniae* but promising in terms of developing new alternative therapeutic strategies against K19-type CRKP infections in the future.

## Introduction

*Klebsiella pneumoniae* is an opportunistic pathogen that causes a variety of infections, including urinary tract infections, bacteremia, pneumonia, and liver abscesses (Wang et al., 2020). Since the first *K. pneumoniae* strain containing the enzyme carbapenemase was identified in 1996, carbapenem-resistant *K. pneumoniae* (CRKP) soon became prevalent, highlighting the issue of lacking available antibiotics and the demand for alternative treatments (Lai et al., 2019). In recent years, the global transmission of CRKP and hypervirulent *K. pneumoniae* strains significantly amplified the damage caused by this pathogen and brought new challenges for effective pathogen control (Zhang et al., 2015). All these factors pointed out that *K. pneumoniae* is a major concern to public health (Effah et al., 2020).

Capsular polysaccharide (CPS), also known as K antigen, is the outermost capsule layer around the *K. pneumoniae* cell, which acts as an essential virulence factor and a defense barrier of *K. pneumoniae* (Effah et al., 2020). It allows the bacterium to survive inside the host by overcoming the protective mechanisms of the immune system. The capsule hinders the bactericidal action of antimicrobial peptides and blocks complement components, preventing complement-mediated killing (Paczosa and Mecsas, 2016; Li J. et al., 2021). It further impairs phagocytosis and opsonophagocytosis of *K. pneumoniae* by immune cells (Opoku-Temeng et al., 2019; Assoni et al., 2021). Until now, at least 134 capsular types have been identified (Volozhantsev et al., 2020). Due to the limited source, high cost of antisera, and large number of serological cross-reactions, the traditional serological typing method is not widely used. The new molecular typing method based on a single *wzi* gene (*wzi* typing) or, for more accurate typing results, the entire genome of the capsular synthesis region (Kaptive typing), has begun to show its advantages over the serological typing method in epidemiological investigations with its excellent typing performance (Zhou et al., 2016; Lam et al., 2022).

Phage-coded capsular depolymerase is an enzyme that bacteriophages use to degrade the CPS of their host bacteria. Recently, studies on capsular depolymerases have shown that each of them is highly specific to a particular CPS layer, demonstrating that they can be used as a simple and economical method to differentiate capsular types, and act as an antibacterial agent for *K. pneumoniae* strains of their corresponding capsular types (Pan et al., 2019). Such characteristics of capsular depolymerases have drawn great attention as they have great potential in defeating CRKP. For example, depolymerases can increase *K. pneumoniae*’s susceptibility to gentamicin at a lower concentration (Bansal et al., 2014); Depolymerase Dpo42 and Dpo43 of K47-type *K. pneumoniae* make host bacteria fully susceptible to the killing effect of serum complement (Liu et al., 2020; Volozhantsev et al., 2020); Depolymerase KP32gp37 and KP32gp38 of K3-type and K21-type *K. pneumoniae*, respectively, can efficiently decrease *K. pneumoniae* resistance to phagocytosis by macrophages (Majkowska-Skrobek et al., 2018). Till now, depolymerases targeting 23 *K. pneumonia* capsular types, namely, K1, K2, K3, K5, K8, K11, K13, K21, K23, K25, K30, K35, K47, K56, K57, K63, K64, K69, KN1, KN2, KN3, KN4, and KN5, have been identified and reported (Wu et al., 2019; Liu et al., 2020; Squeglia et al., 2020; Volozhantsev et al., 2020; Dunstan et al., 2021; Gorodnichev et al., 2021; Li J. et al., 2021; Pertics et al., 2021).

The number of hospitalized and agriculturally transmitted multi-drug resistant *K. pneumoniae* strains has shown a rising trend in the past few years in China (Xu et al., 2016; Yang et al., 2021). K19 *K. pneumoniae* serotype has brought our attention as it was discovered as the second most prevalent serotype in a local hospital CRKP sample population (*n* = 348) in Shanghai, China (Zhang et al., 2020) and the third most prevalent serotype in a population (*n* = 592) of local Chinese adults and overseas Chinese in Japan, Malaysia, Singapore, Thailand, and Vietnam (Volozhantsev et al., 2020). However, no report has been published on any depolymerase that specifically targets K19-type *K. pneumoniae* strains. In this study, we identified a new phage-derived depolymerase targeting specifically K19-type *K. pneumoniae* strains for the first time. Our results demonstrated that this depolymerase presented great potential in advancing capsular typing and treatments of K19-type CRKP infections.

## Materials and Methods

### Bacterial Strain Isolation and Identification

Twenty-nine *K. pneumoniae* strains collected from Renji Hospital, Shanghai Jiao Tong University School of Medicine, and Huashan Hospital, Fudan University (listed in Table 1) were used in this study. All strains were identified by the matrix-assisted laser desorption ionization time-of-flight mass spectrometry (Bruker Diatonic GmbH, Bremen, Germany; D’Andrea et al., 2017). Each *K. pneumoniae* strain was determined as CRKP with resistance to imipenem or meropenem. The antimicrobial susceptibility was determined using the Kirby-Bauer (K-B) method. The capsular types of these *K. pneumoniae* strains were determined by *wzi* gene sequencing according to Brisse et al. (2013). The viable bacterial count was determined using LB agar (1.5% w/v) plates. All strains were cultivated in Luria-Bertani broth (LB, Sangon Biotech, Shanghai, China) at 37^°^C and stored at –80^°^C in 50% glycerol. *K. pneumoniae* strain 6570 was used for the phage isolation, the serum assay and the establishment of the infection model.

**TABLE 1:** Host spectrum of phage SH-KP156570 and depolymerase K19-Dpo41 against 29 *K. pneumoniae* strains.

Isolate no.	Capsular type	SH-KP156570		K19-Dpo41
		Plaques	Halos	
6570^1^*, 6778^1^, 7124^1^, 7321^1^, 7485^1^, 7696^1^, 7762^1^, 8144^1^, 8576^1^, 18–199^2^, 18–201^2^, 18–371^2^, 19–79^2^, 19–567^2^, 19–788^2^, 19–791^2^, 19–831^2^, 19–853^2^, 19–854^2^, 19–855^2^	K19	+	+	+
5080^1^	K1	–	–	–
5146^1^, 5170^1^, 7956^1^	K2	–	–	–
6371^1^, 6408^1^	K20	–	–	–
8031^1^	K47	–	–	–
5169^1^	K57	–	–	–
6089^1^	K64	–	–	–

*The capsular type of K. pneumoniae strains were determined by wzi genotyping. –, no lysis; +, showed plaques or haloes.*

*^1^Isolated from patients at Renji Hospital in 2019 in Shanghai, China.*

*^2^Isolated from patients at the Huashan Hospital, affiliated with Fudan University in Shanghai, China.*

*Host strain of phage SH-KP156570.

### Phage Isolation, One-Step Growth Curve, and Host Spectrum Analysis

Phage SH-KP156570 was isolated from a raw sewage collected at Ruijin Hospital, Shanghai Jiao Tong University School of Medicine by using *K. pneumoniae* strain 6570 as the host bacterium. After centrifugation, the supernatant was filtered with a 0.22 μm filter (Millex-GP Filter Unit; Millipore, United States) and dripped on a double-layer agar plate covered with *K. pneumoniae* strain 6570. The lytic phage was then purified according to Pires’s method with minor modifications (Pires et al., 2011). The purified phage named SH-KP156570 was stored in SM buffer (100 mM NaCl, 8 mM MgSO_4_ 7H_2_O, 50 mM Tris–HCl, pH = 7.5) at 4^°^C.

The one-step phage growth curve was generated as described previously with some modifications (D’Andrea et al., 2017). Briefly, Phage SH-KP156570 and *K. pneumoniae* strain 6570 were mixed at an MOI = 0.005. After bathing at 37^°^C for 5 min, the mixture was centrifuged, resuspended in 3 mL liquid LB medium. Samples were taken for every 3 min and dripped on the double-layer agar plate at a proper dilution. The number of bacteriophage plaque was counted overnight and the titer of bacteriophage was calculated. The number of plaque-forming units (PFU) was then calculated for 1 mL of the concentrated suspension. The latency period was defined as the time between infection and the shortest incubation time, allowing for the production of phages. The burst size was calculated as the ratio of the final count of released phage particles to the initial count of infected bacterial cells during the latent period. Independent experiments were repeated three times.

Host range analysis of phage SH-KP156570 was performed by the spot test using the strains listed in Table 1. In short, 400 μL log-phase bacterial culture was fully mixed with 4 mL 0.5% agar LB and laid on 1.5% agar LB medium. After the upper agar was solidified, the 5 μL purified phage suspension was spotted onto the plate and incubated overnight at 37^°^C to allow plaques to develop.

### Genomic DNA Sequencing and Annotation

The genomic DNA of SH-KP156570 was extracted using the standard protocol as previously described (Guo et al., 2017). In brief, the phage in SM buffer was treated with 1 μg/mL DNaseI and RNaseA (Sigma-Aldrich, United States) at 37^°^C for 1 h, subsequently 25 mmol/mL ethylenediaminetetraacetic acid. Finally, the concentrated phage DNA was extracted using the UNlQ-10 Column Virus Genomic DNA Isolation Kit (Sangon Biotech, Shanghai, China) and sent to Shanghai Personalbio Biotechnology Co. Ltd for genome sequencing with Illumina high-throughput sequencing platform (Illumina Hiseq 3000). SOAP denovo2 software was used for genome assembly using optimized parameters. Data assembly was proceeding after adapter contamination removing and data filtering by using AdapterRemoval (Lindgreen, 2012) and SOAPec (Luo et al., 2012). The filtered reads were assembled by SPAdes (Bankevich et al., 2012) and A5-miseq (Coil et al., 2015) to constructed scaffolds and contigs. GeneMark was used to predict and analyze the open reading frames (ORFs) of the phage genome. Gene annotation was completed by using the NCBI website^1^. Function annotation was completed by blast search against different databases, including NR (Non-Redundant Protein Database; Blake and Cohen, 2001), Gene Ontology (Conesa and Götz, 2008), Kyoto Encyclopedia of Gene and Genomes (Moriya et al., 2007), Cluster of Orthologous Groups of proteins (Powell et al., 2014), and Swissprot. CGview (Stothard and Wishart, 2005) was used to give an overview of the genome information. The results of the HHpred database^2^ were used to locate the gene encoding polysaccharide depolymerase [Uniclust was chosen as the multiple sequence alignment (MSA) generation method, and three was chosen as the maximal number of MSA generation steps].

### Cloning, Expression, and Purification of the Recombinant Depolymerase

The ORF41 gene was amplified from the DNA of the purified phage SH-KP156570 using primers 6570-ORF41-F (5′-CAGCAGCAGACGGGAGGATCCATGTCCACGATTACACA ATTC-3′) and 6570-ORF41-R (5′-CTCGAGTGCGGCCG CAAGCTTTTAGTTACTTCTCTCTTCAGC-3′). The 2292-base PCR amplification product was cloned into the N-terminal 6 × His labeled pSUMO3 expression vector (LifeSensors, United States) *via Bam*HI and *Hin*dIII sites (New England Biolabs). The recombinant plasmid was verified by DNA sequencing and transformed into *E. coli* BL21 (DE3). The BL21 cells were cultivated to OD_600_ = 0.8. Then, the BL21 cells were induced with 0.1 mM isopropyl-β-D-thiogalactopyranoside (IPTG, Sangon Biotech, China). The mixture was centrifuged and the supernatant was discarded. The sediment was resuspended with lysis buffer [20 mM Tris–HCl (pH = 8.0), 0.5M NaCl, 10% glycerol] and phenylmethylsulfonyl fluoride (PMSF, Sangon Biotech, China) to a final concentration of 0.1 mg/mL. After centrifuged, the supernatant was collected. The Ni-NTA column (GE Healthcare, United States) was prerinsed using lysis buffer. After protein binding to Ni-NTA column, it was eluted with a gradient of 20 and 40 mM imidazole, and finally eluted with 300 mM imidazole. Then digested by SUMO protease (LifeSensors, United States). The purified depolymerase was confirmed by SDS-PAGE electrophoresis and named K19-Dpo41.

### Purification of the Capsular Polysaccharide

*Klebsiella pneumoniae* strain 6570 was cultured in fresh TSB medium at 37^°^C for 5 days. The purification of CPS was performed according to the method from previous studies with some modifications (Gorodnichev et al., 2021; Li J. et al., 2021). Firstly, 60 μL of formaldehyde solution (36.5%) was added to 10 mL of bacterial culture and incubated 100 rpm at room temperature for 1 h. Subsequently, 1M NaOH was added to the system, with agitation at room temperature for 3 h. Cell suspensions were centrifuged at 16,800 *g* for 1 h at 4°C (Beckman, JA-25.50, United States). The supernatant was filtered through a 0.22 μm filter and dialyzed overnight in ddH_2_O with a 12–14 KD MWCO membrane (Thermo Scientific, United States). Then, the cationic detergent cetyltrimethylammonium bromide was added (0.5% w/v) to precipitate polysaccharide. Dissociate with different concentrations of CaCl_2_ and centrifuge the supernatant. Selective precipitation with 20 and 50% ethanol and washed with NaCl. Next, the sample was subjected to Capto adhere chromatography after ultrafiltration with a 30 KD membrane. The final sample was filtered through a 0.22 μm filter. Finally, the CPS obtained by dialysis was dried and weighed.

### Depolymerization Activity Assay of K19-Dpo41 on *K. pneumoniae* Strain 6570 Capsular Polysaccharide

The activity of K19-Dpo41 was assessed by spot test (Khan Mirzaei and Nilsson, 2015). After *K. pneumoniae* strain 6570 was poured onto a LB agar, a serial 5 μL polysaccharide depolymerase in different concentration was spotted on the plate and incubated overnight at 37°C. The K19-Dpo41 activity was monitored by observing the formation of translucent halo zones. In addition, the sensitivity of other *K. pneumoniae* strains to K19-Dpo41 was also determined by single-spot assay (Wu et al., 2019).

Size exclusion chromatography-High performance liquid chromatography (SEC-HPLC) was used to determine the depolymerase activity of K19-Dpo41 on the CPS. The purified CPS was dissolved in 50 mM Na_2_HPO_4_ (pH = 7.0) to a final concentration of 0.5 mg/mL and incubated with K19-Dpo41 (10 μg/mL) or ddH_2_O at 37^°^C for 30 min. The reactions were terminated by heating at 100°C for 10 min. The mixture was analyzed by a HPLC system (Waters, Milford, MA, United States) equipped with a TSKgel G5000 PWxl column (inner diameter 7.8 mm × 300 mm; Tosoh Corporation Bioscience, Tokyo, Japan) and a refractive index detector (Waters, Milford, MA, United States). The column was run with the mobile phase of the phosphate-buffered saline (PBS, pH = 7.0) at 1 mL/min.

### Serum Resistance Assay

The serum resistant assay was performed as previous studies (Liu et al., 2020; Li J. et al., 2021). *K. pneumoniae* strain 6570 at a concentration of 5 × 10^4^ CFUs were suspended in PBS, treated with either K19-Dpo41 or K64-ORF41 (a depolymerases specific to *K. pneumoniae* K64 serotype), respectively, to a final concentration of 100 μg/mL. After a pretreatment at 37°C for 1 h, the suspension was mixed at a 1:3 v/v ratio with active or heat-inactivated human serum obtained from healthy volunteers. A control without depolymerase was also set up for significance. The mixture in final volume of 100 μL was incubated for 1 h at 37^°^C, and then 5 μL aliquots were removed, diluted and cultured on 1.5% LB agar plate for colony enumeration. Each test was performed at least in three independent experiments. Comparisons between any two experimental groups were made by the two-sample Student’s *t*-test. *P* < *0.05* was considered statistically significant.

### *Galleria mellonella* Larvae Infection Model

Wax moth larvae (*G. mellonella*) were obtained from Tianjin Huiyude BioTech, China. The operating procedure was carried out in accordance with the previous studies (Majkowska-Skrobek et al., 2016; Oliveira et al., 2019; Gorodnichev et al., 2021; Shahed-Al-Mahmud et al., 2021). In short, *K. pneumoniae* strain 6570 from overnight culture were grown in fresh LB at 37°C to exponential phase, harvested (10,000 rpm, 10 min), washed with PBS and suspended in PBS to a concentration of 5 × 10^6^ CFU/mL. Larvae were inoculated with 10 μL bacterial suspension containing 5 × 10^4^ CFUs into the last proleg using 25 μL syringe. The anti-virulence effect of enzyme on *K. pneumoniae* strain 6570 was estimated by inoculated larvae with either K19-Dpo41 administered at 5 min or 30 min after bacterial infection. Larvae injected with either PBS or 5 × 10^4^ CFUs of *K. pneumoniae* strain 6570 were included as control groups. After inoculation, caterpillars were kept at 37°C in the dark for 5 days. The results were expressed as the percentage survival rate estimated on the basis of the touch-provoked motility at each day post injection. For each option, at least three independent experiments were performed (10 larvae per trial). The data was presented as the average of three experiments.

### Statistics

GraphPad Prism (version 8, GraphPad Software, United States) software was used for the statistical analysis. The data were tabulated as mean ± SD. In the serum resistance analysis, comparisons between any two experimental groups were made by the two-sample Student’s *t*-test. In the *Galleria mellonella* larvae infection model, survival curves were plotted using the Kaplan–Meier method, and the survival analysis was performed by using the log-rank Mantel-Cox test. *P <* 0.05 was considered to be statistically significant.

### Ethics Statement

The strain specimens were collected with the written and informed consent of the patients. The conduct and procedures involved in the present work were approved by the Ethics Committee of Shanghai Jiao Tong University School of Medicine.

## Results

### Antimicrobial Resistance and Capsular Genotypes of *K. pneumoniae* Strains

All 29 clinical isolates were identified as CRKP and resistant to a variety of antibiotics, including cefuroxime (CXM, 28/29), amikacin (AMK, 15/29), piperacillin-tazobactam (TZP, 28/29), levofloxacin (LVX, 27/29), ciprofloxacin (CIP, 27/29), ceftazidime (CAZ, 29/29), cefoperazone sodium Shubatan (CSL, 27/29), cefepime (FEP, 29/29), Imipenem (IPM, 19/29), meropenem (MEM, 28/29), trimethoprim-sulfamethoxazole (SXT, 17/29), and aztreonam (ATM, 27/29). Twenty-nine *K. pneumoniae* were genotyped by *wzi* sequencing. Twenty of them were Type K19, others were K1, K2, K20, K47, K57, and K64 (Table 1).

### Bacteriophage SH-KP156570 Is Specific for K19-Type *K*. *Pneumoniae*

**FIGURE 1:**
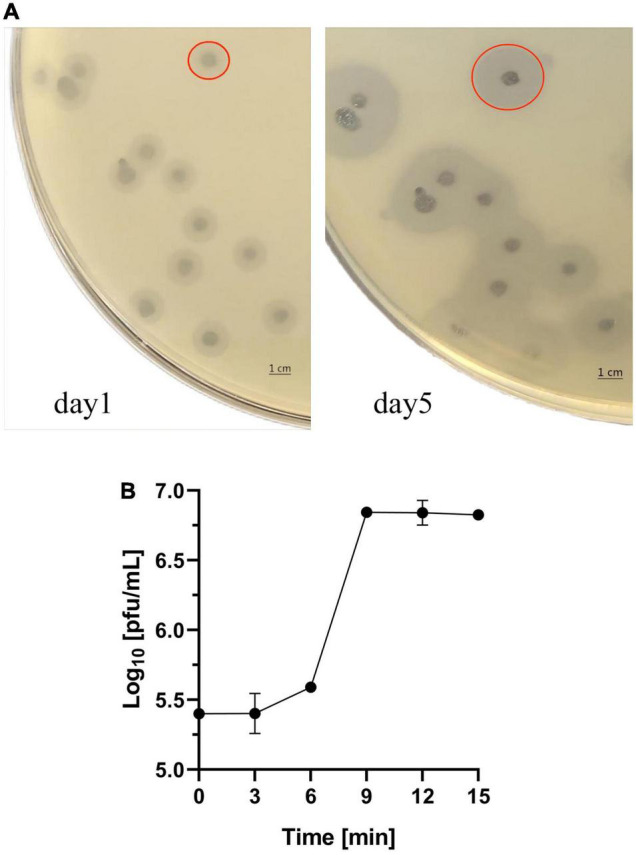
Characterization of Phage SH-KP156570. **(A)** Interaction between the phage and *K. pneumoniae* strain 6570. Plagues were formed on the plate. Faint halos could be observed around the plaque on day 1 and the size of the faint halos increased by day 5. The red circle in the picture represented the size of the plaque and halo. **(B)** One-step growth curve of SH-KP156570. The burst size was calculated as the ratio of the final count of phage particles to the bacterial cells. Phage concentration in PFU/mL as a function of time post-infection was plotted. Error bars represent mean ± SD.

*Klebsiella pneumoniae* strain 6570 and the sewage collected from hospital were co-cultured overnight and phage SH-KP156570 was successfully isolated. The phage was spread over *K. pneumoniae* strain 6570 to form a double-layer agar plate and cleared plaques formations and faint halos were observed. The size of the faint halos increased over time (Figure 1A), which we suspected indicated the presence of a phage-derived depolymerase based on our previous study (Li J. et al., 2021). To understand the relationship between SH-KP156570 and capsular types, we performed a host spectrum analysis of SH-KP156570 on all 29 strains using spot tests. Faint halos and plague were observed over time for all twenty K19-type strains, while absences of plaque or halo were observed for nine non-K19-type strains (Table 1). The results indicated that phage SH-KP156570 targeted K19 CPS exclusively.

### Determination of the Life Cycle of SH-KP156570

The life cycle of SH-KP156570 was revealed with a one-step growth curve. The phage had a short latent period of 6 min followed by a rise period of the phage progeny until a stationary phage was reached at 9 min (Figure 1B). The burst size was about 80 PFU per infected cell.

### Genomic DNA Sequencing and Annotation of Phage SH-KP156570

**FIGURE 2:**
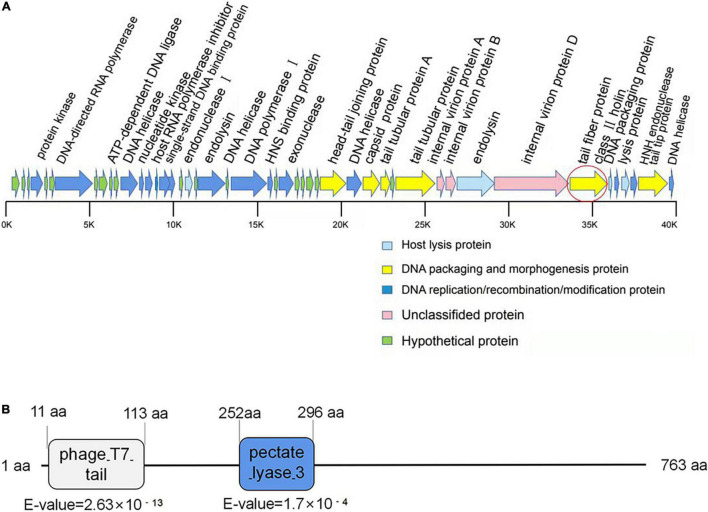
Bioinformatic analysis of the genome of phage SH-KP156570. **(A)** Gene map of the phage SH-KP156570. Predicted ORFs were shown. The linear map was based on nucleotide sequences of the whole genome. The red circle denoted ORF41, the gene encoding the putative polysaccharide depolymerase. **(B)** Bioinformatic analysis of the putative depolymerase of the phage SH-KP156570. The result showed a 763 aa protein with two conserved domains: phage T7 (residue 11–113) and pectate lyase domain (residue 252–296) by using BLASTp and HHpred. ORF41 was determined and identified as protein K19-Dpo41.

A single contiguous sequence of 38,667 bp was *de novo* assembled from 8,102,358 raw sequencing reads with more than 30,330 × coverage. Hence, the phage SH-KP156570 had a genome size of 38,667 bp with a GC content of 50.85%. The whole genome sequence of the SH-KP156570 was deposited in National Genomics Data Center^3^ with the accession number (GWHBGZN00000000). We identified 47 ORFs. Genes encoding putative functional protein were assigned to 29 of the 47 ORFs (61.7%). The average size of these ORFs was 851 bp. Phage-encoded proteins could be divided based on their functions: DNA packaging and morphology-related proteins (6), DNA replication/recombination/modification proteins (16), host lytic proteins (4), and unknown proteins (3). The remaining genes were encoded as hypothetical proteins. Among the 29 functional annotated genes, genes involved in DNA processing and packaging machinery, structural and lytic proteins, DNA replication, DNA recombination, and repair molecular mechanisms were identified. No putative integrase genes, virulence factors, toxins, or antimicrobial resistance genes were found in the genome of SH-KP156570 (Figure 2A).

Gene ORF41 (2292bp), coding for the tail fiber protein, was predicted to be a depolymerase-encoding gene. The N-terminal conserved domain (11 to 113 aa) of ORF41 protein showed a high similarity with the N-terminal sequence of T7 phage, while the central region (252 to 296 aa) showed a high similarity with the pectate lyase (Figure 2B). This ORF protein contained a β-helical pectin lyase domain, and had a 28% identity to the tail spike protein TSP3 from *Escherichia coli* phage CBA120 (a lipopolysaccharide lyase of *E. coli*; Greenfield et al., 2019). These results suggested that the ORF41 protein might be a polysaccharide depolymerase in phage SH-KP156570.

### Depolymerization Activity of Recombinant Depolymerase K19-Dpo41

**FIGURE 3:**
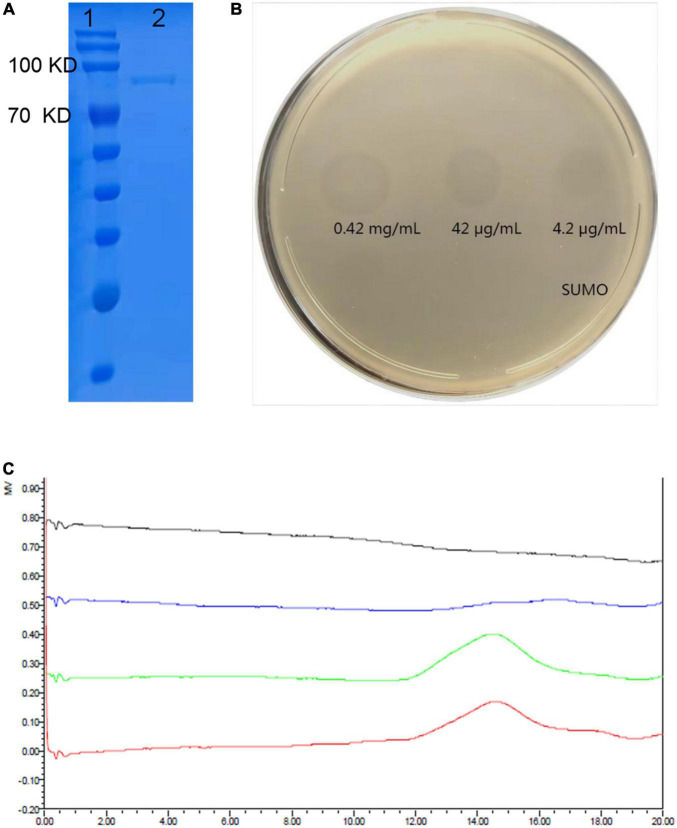
Expression and depolymerization activity of recombinant depolymerase K19-Dpo41. **(A)** 10% SDS-PAGE gel analysis on the purity of K19-Dpo41. The purity of the purified K19-Dpo41 was more than 95%. Lane 1, protein marker; lane 2, purified K19-Dpo41. **(B)** Spot test of purified depolymerases K19-Dpo41 on *K. pneumoniae* strain 6570 lawn. Aliquots of serial dilutions of K19-Dpo41 was spotted onto a plate containing the *K. pneumoniae* strain 6570. SUMO protein was served as a negative control. **(C)** Size exclusion chromatography-High performance liquid chromatography (SEC-HPLC) analysis of capsular polysaccharide (CPS) treated with K19-Dpo41. Red line, purified untreated CPS; green line, CPS incubated with 10 μg/mL SUMO at 37°C for 30 min; blue line, CPS treated with 10 μg/mL K19-Dpo41 at 37°C for 30 min; and gray line, K19-Dpo41 incubated at 37°C for 30 min.

The ORF41 gene of phage SH-KP156570 was cloned into the pSUMO3 expression vector. The recombined K19-ORF41-pSUMO3 was expressed and purified by Ni-NTA column. The purity of the purified K19-Dpo41 (about 84 kDa) was more than 95% confirmed by SDS-PAGE gel analysis (Figure 3A). The purified recombinant depolymerase K19-Dpo41 were diluted to different concentrations (4.2 μg/mL∼0.42 mg/mL) for the spot test to assess the polysaccharide depolymerization activity. Clear halos were observed at different K19-Dpo41 concentrations as low as 4.2 μg/mL (Figure 3B). The SEC-HPLC results confirmed the depolymerization of K19-Dpo41 against the CPS of *K. pneumoniae* strain 6570. The untreated CPS showed a single peak with a retention time from 12 to 16 min, however, after incubation with K19-Dpo41 at 37°C for 30 min, the peak of CPS disappeared, demonstrating that CPS was degraded by K19-Dpo41 (Figure 3C).

The activity of K19-Dpo41 was further tested on all 29 *K. pneumoniae* strains using the spot assay. On plates incubated with K19-type *K. pneumoniae* strains, the recombinant protein K19-Dpo41 generated a translucent halo zone (Table 1). Meanwhile, no translucent spots formed on any non-K19-type *K. pneumoniae* strains as expected. The results showed that depolymerase K19-Dpo41, same as the parental phage SH-KP156570, exhibited specificity to K19-type *K. pneumoniae*.

### K19-Dpo41 Increased the Susceptivity of *K. pneumoniae* Strain 6570 to Serum Killing

**FIGURE 4:**
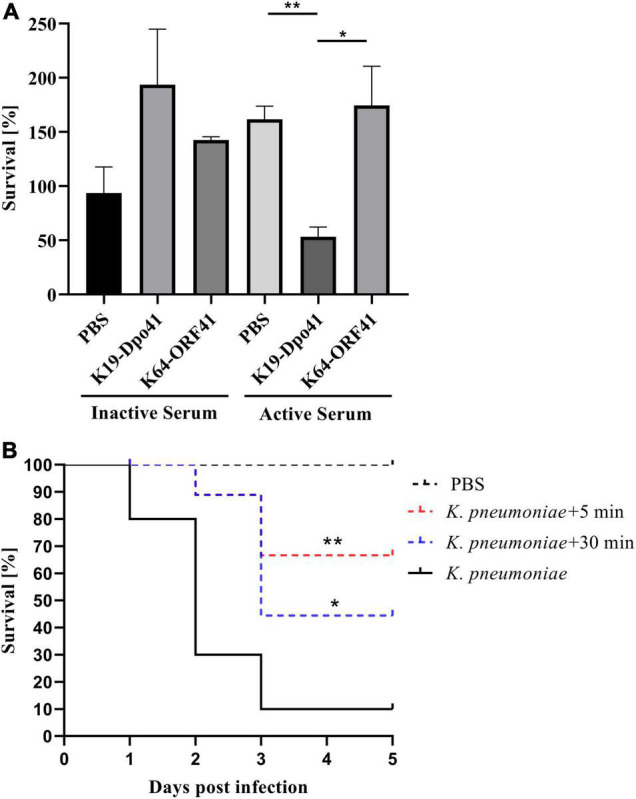
Anti-*k. pneumoniae* infection effect of K19-Dpo41. **(A)** Effect of K19-Dpo41 on bacterial susceptibility to serum killing. *K. pneumoniae* strain 6570 treated with K19-Dpo41 were incubated with active or heat-inactivated human serum obtained from healthy volunteers for 1 h at 37^°^C. A control group with no K19-Dpo41 was included. A specificity group with K64-ORF41, another depolymerase specifically targeting the CPS of K64-type *K. pneumoniae*, was also included. The survival rate was defined as the average percent survival of bacteria relative to the initial population. The experiment was repeated three times. Error bars represent mean ± SD. The individual survival rates of each group were compared by Student’s *t* test. **P < 0.01, *P < 0.05. **(B)** Effect of K19-Dpo41 on larvae survival rate in a *Galleria mellonella* larvae infection model. Black solid line, Larvae injected with 5 × 10^4^ CFUs *K. pneumoniae* strain 6570; red dotted line, K19-Dpo41 (final concentration, 2 μg per larva) administered 5 min after 5 × 10^4^ CFUs *K. pneumoniae* strain 6570 inoculation; blue dotted line, K19-Dpo41 (final concentration, 2 μg per larva) administered 30 min after 5 × 10^4^ CFUs *K. pneumoniae* strain 6570 inoculation; black dotted line, Larvae injected with PBS. The mortality were monitored for 5 days (*n* = 10). Results are the means of three independent experiments. Survival curves were plotted using the Kaplan–Meier method, and differences in survival were calculated by using the log-rank test (GraphPad). **P<0.01 and *p<0.05, mean a significant difference to the *K. pneumoniae* strain 6570 infected group.

We assessed the bactericidal effect of serum after depolymerase treatment on *K. pneumoniae* strain 6570. After 1 h of incubation, the survival rate of bacteria treated with K19-Dpo41 decreased by around 70% compared with those of untreated bacteria and bacteria treated with K64-ORF41, a depolymerase specific for K64-type *K. pneumoniae*, demonstrating that K19-Dpo41 could improve the susceptivity of K19 *K. pneumoniae* to serum killing (Figure 4A).

### Anti-*k. pneumoniae* Infection Efficacy of K19-Dpo41 Treated *K. pneumoniae* in a *Galleria mellonella* Larvae Infection Model

We also evaluated the anti-*k. pneumoniae* infection efficacy of K19-Dpo41 using *K. pneumoniae* infected *G. mellonella* larvae model. Our results showed that in the *K. pneumoniae* infection group, 90% of the larvae injected with *K. pneumoniae* strain 6570 died within 3 day. In contrast, a single dose of K19-Dpo41 given 30 min after bacteria injection increased the survival rate of the larvae to 50%, and the same dose given 5 min reached to 70%. No mortality of larvae was observed in the group injected with PBS.

## Discussion

*Klebsiella pneumoniae* is a clinically significant organism that has proposed a significant amount of threat to public health (Ashurst and Dawson, 2021). The prevalence of CRKP, one of the deadliest strains of *K. pneumoniae*, is a challenging medical and economical concern that needs to be addressed immediately, especially in countries where infections have become endemic (Karampatakis et al., 2016; Mohammad Ali Tabrizi et al., 2018; Shankar et al., 2018; Effah et al., 2020). Specifically in China, a considerable number of multi-drug resistant *K. pneumoniae* strains have been isolated and identified from hospitalized patients and local agriculture lands from multiple regions in the past few years (Xu et al., 2016; Yang et al., 2021). Among currently known CRKP strains, K19 serotype was determined as one of the most prevalent *K. pneumoniae* serotypes in many isolates in previous studies (Volozhantsev et al., 2020; Zhang et al., 2020). It was also found to be strongly associated with the *bla*_KPC–2_ antimicrobial resistance gene (Liao et al., 2021). Therefore, the development of a novel, effective therapeutic approach to overcome K19-type *K. pneumoniae* is urgently needed.

Recently, phage-derived capsular depolymerases that degrade the distinct CPS of *K. pneumoniae* have been discovered as promising alternatives to treat *K. pneumoniae* infections. Compared to using phages that directly lyse bacteria, depolymerase-mediated treatments show higher specificity (Pan et al., 2017). They are also found to be safer since phage therapies may result in the development of bacterial resistance to phages and new antimicrobial resistance, which would not be a concern when using depolymerases (Loc-Carrillo and Abedon, 2011; Principi et al., 2019). So far, 34 distinct depolymerases that targeted a range of 22 *K. pneumoniae* capsular types were discovered (Wu et al., 2019; Liu et al., 2020; Squeglia et al., 2020; Volozhantsev et al., 2020; Dunstan et al., 2021; Gorodnichev et al., 2021; Li J. et al., 2021; Pertics et al., 2021). These depolymerases had great potential for capsular typing of *K. pneumoniae*, and they were found to be effective in raising the susceptivity of bacteria to serum killing (Pan et al., 2019; Li J. et al., 2021). However, depolymerases distinctly targeting K19-type CRKP, an epidemiologically significant CRKP strain in China, have never been reported. Our study was the first that identified a new phage-derived depolymerase (K19-Dpo41), which specifically degrades the capsule of K19-type *K. pneumoniae*.

In our previous study, we confirmed that using capsular depolymerase, which directly identified the composition and structure of polysaccharides, could trace the mutations in the CPS synthesis region of the *K. pneumoniae* genome. It was a more reliable capsular typing method compared to the *wzi* genotyping (Li J. et al., 2021). The depolymerase K19-Dpo41 we isolated and characterized in this study had high specificity for K19-type *K. pneumoniae*, which allowed an accurate typing of K19-serotype *K. pneumoniae*.

The antibacterial effect of *K. pneumoniae* phage-derived depolymerases made them a potential therapeutic alternative for treating *K. pneumoniae* infections (Blundell-Hunter et al., 2021; Gorodnichev et al., 2021; Li M. et al., 2021). Unlike phages that directly lysed the host bacteria, depolymerases only cleaved the CPS so that the bacteria became more sensitive to the activities of the surrounding complement and phagocytes, activating the host innate immune responses (Li J. et al., 2021). To assess the anti-virulence effect of K19-Dpo41, we conducted the serum complement-mediated killing assay and used *G. Mellonella* Larvae infection model to see the depolymerase’s effect on the susceptivity of the K19-type *K. pneumoniae* strain 6570. The serum-killing assay showed that the survival rate of bacteria pretreated with K19-Dpo41 decreased significantly compared to untreated bacteria or bacteria pretreated with non-specific depolymerase K64-ORF41, a depolymerase that was found not targeting and degrading K19 but instead K64 capsular serotype (Figure 4A). K19-Dpo41 also significantly reduced the mortality of *G. mellonella* larvae infected with K19-type *K. pneumoniae* strain 6570 (Figure 4B). These results suggested that K19-Dpo41 could become a promising agent in treating K19-type *K. pneumoniae* infections.

In conclusion, our study demonstrated that K19-Dpo41, a novel depolymerase derived from phage SH-KP156570, was able to degrade the capsule of K19-type *K*. *pneumoniae*, promoted the susceptivity of the bacteria to serum complement lysis, as well as effectively increased the survival rate of *G. mellonella* larvae in an *in vivo* infection model. This depolymerase and its enzymatic activity we characterized would surely be found to have beneficial applications relative to the treatment, prevention, and control of severe CRKP infections, and for more accurate, efficient capsular typing.

## Data Availability Statement

The datasets presented in this study can be found in online repositories. The names of the repository/repositories and accession number(s) can be found below: https://ngdc.cncb.ac.cn/gwh, GWHBGZN00000000.

## Ethics Statement

Written informed consent was obtained from the individual(s) for the publication of any potentially identifiable images or data included in this article.

## Author Contributions

YH, YW, MG, HL, QL, and PH drafted the manuscript and performed the data analysis. YH, YW, and MG planned and performed experiments. YH, YW, MG, QL, and PH were responsible for experimental design. All authors have read and revised the manuscript.

## Conflict of Interest

The authors declare that the research was conducted in the absence of any commercial or financial relationships that could be construed as a potential conflict of interest.

## Publisher’s Note

All claims expressed in this article are solely those of the authors and do not necessarily represent those of their affiliated organizations, or those of the publisher, the editors and the reviewers. Any product that may be evaluated in this article, or claim that may be made by its manufacturer, is not guaranteed or endorsed by the publisher.

## Footnotes

1
https://blast.ncbi.nlm.nih.gov/Blast.cgi

2
https://toolkit.tuebingen.mpg.de/tools/hhpred

3
https://ngdc.cncb.ac.cn/gwh
